# Using primary health care (PHC) workers and key informants for community based detection of blindness in children in Southern Malawi

**DOI:** 10.1186/1478-4491-10-37

**Published:** 2012-09-27

**Authors:** Khumbo Kalua, Ruby Tionenji Ng’ongola, Frank Mbewe, Clare Gilbert

**Affiliations:** 1 Department of Ophthalmology, University of Malawi, College of Medicine, Blantyre, Malawi; 2 Blantyre Institute for Community Ophthalmology (BICO), Lions Sight First Eye Hospital, Blantyre, Malawi; 3 London School of Hygiene and Tropical Medicine (LSHTM), London, UK

**Keywords:** Key informants, Health Surveillance Assistants, Primary eye care, Blindness, Children, Malawi, Community

## Abstract

**Background:**

There is great interest in providing primary eye care (PEC) through integration into primary health care (PHC). However, there is little evidence of the productivity of PHC workers in offering primary eye care after training and integration, and there is need to compare their effectiveness to alternative methods. The current study compared the effectiveness of trained Health Surveillance Assistants (HSAs) versus trained volunteer Key Informants (KIs) in identifying blind children in southern Malawi.

**Methods:**

A cluster community based study was conducted in Mulanje district, population 435 753. Six clusters each with a population of approximately 70 000 to 80 000, 42% of whom were children were identified and randomly allocated to either HSA or KI training. From each cluster 20 HSAs or 20 KIs were selected for training. Training emphasized the causes of blindness in children and their management, and how to identify and list children suspected of being blind. HSAs and KIs used multiple methods (door to door, school screening, health education talks, village announcements, etc.) to identify children. Using the World Health Organization (WHO) estimates (eight blind children per 10 000 children); approximately 144 to 162 blind children were expected in the chosen clusters. Listed children were brought to a centre within the community where they were examined by an ophthalmologist and findings recorded using the WHO form for examining blindness in children.

**Results:**

A total of 59 HSAs and 64 KIs were trained. HSAs identified five children of whom two were confirmed as blind (one blind child per 29.5 HSAs trained). On the other hand, the KIs identified a total of 158 children of whom 20 were confirmed blind (one blind child per 3.2 KIs trained). More blind boys than girls were identified (77.3% versus 22.7%) respectively.

**Conclusion:**

Key Informants were much better at identifying blind children than HSAs, even though both groups identified far fewer blind children compared with WHO estimates. HSAs reported lack of time as a major constraint in identifying blind children. Based on these findings using HSAs for identifying blind children would not be successful in Malawi. Gender differences need to be addressed in all childhood blindness programs to counteract the imbalance.

## Background

According to the World Health Organization (WHO), Sub-Saharan Africa (SSA) continues to experience severe shortages of human resources for health from primary through to tertiary levels [1]. The provision of adequate eye services is affected by this human resource crisis and there have been discussions concerning the use of general rather than the non-available specialized eye health workers to improve eye health service delivery. At the same time, renewed emphasis has been placed on the vital importance of primary health care as the building block upon which health care systems can be built [2,3].

Primary health care (PHC) is essential health care that is universally acceptable and accessible to individuals and families in the community and where there is full community participation. The overall goal of PHC is to promote and protect the health of all individuals. The core principles of PHC are community participation, equity, inter-sectoral collaboration, sustainability and appropriate affordable technology. The eight key components of PHC are water and sanitation, food and nutrition, immunization against major childhood diseases, maternal and child health (MCH), prevention and control of locally endemic diseases, treatment for common diseases and injuries, health education about prevention/control of important diseases, and provision of essential drugs [4].

Primary eye care (PEC) involves provision of eye services at the community level and these services include eye health promotion within the community, case detection, diagnosing eye problems, providing initial treatment and referring cases, where appropriate. PEC aims to prevent unnecessary causes of blindness in adults and children, and to identify those who need treatment and those who need rehabilitative services. PEC is particularly important in regard to control of blindness in children where late case detection and appropriate treatment can have long lasting consequences on the child and family.

In terms of eye care delivery, there is interest in integrating PEC into PHC by increasing the knowledge, skills and support of PHC workers [5,6]. This approach has the potential to improve trachoma control, to prevent corneal blindness in children from vitamin A deficiency and measles infection, to provide treatment for common eye infections, such as conjunctivitis and injuries, and to identify and refer individuals who need sight restoring procedures such as surgery for cataract or spectacles for refractive error.

The prevalence of blindness in children varies from region to region depending on socioeconomic development and ranges about 3 blind children per 10 000 in developed regions to 15 per 10 000 in less developed regions [7]. Preventable causes of eye diseases and blindness are more prevalent in less developed regions, such as Malawi. The WHO prevalence estimate of blindness in children in Malawi is 8 per 10 000 children [7]. This means for every one million population where approximately 42% are children aged <16 years, there will be approximately 336 children who are blind, from all causes except refractive error. The causes of blindness in children include treatable conditions such as cataract and corneal ulcers, and other non-avoidable causes such as retinal and cortical diseases. PEC is needed to detect children who have treatable causes, such as congenital cataracts, as early as possible, as early detection and surgery is associated with better visual outcomes.

Over the last few years emphasis has been placed on developing the PHC system in Malawi (population 14 000 000), with more than 10 500 PHC workers known as Health Surveillance Assistants (HSAs) trained. HSAs are high school leavers who attend a three-month course and are then deployed by the Ministry of Health to provide preventive, curative and rehabilitative services in the community with a focus on MCH. Each HSA covers a population of 1000 to 1500 population (1 to 2 villages) and there are approximately 10 to 20 HSAs providing services within a catchment area of each health centre. With the recent calls by WHO to strengthen health systems [8], and the renewed emphasis on PHC [3,9], HSAs have become a vital component of health service delivery in Malawi [10]. Indeed the goal of achieving the millennium development goals (MDGs) is highly dependent on the services provided by the HSAs.

Integrating PEC into their work has the potential to improve access to eye care, particularly in rural areas. However, there is still very little information in Africa regarding how PEC should be implemented to improve and sustain eye service delivery [11-13]. Most previous efforts have concentrated on imparting knowledge and skills through training of health workers in PEC and some studies have shown that providing support and supervision in addition to training is more likely to lead to better outcomes than training alone [13]. A recent review of the effectiveness of PEC concluded that before PEC is fully accepted in Africa, there was a need to generate more information on its effectiveness and limitations [12]. Some of the challenges to implementing PEC in Africa have been lack of clarity regarding the definition and the scope of PEC and who should be trained to deliver which elements and what would constitute the minimum skills required.

In the absence of evidence on integration and the effectiveness of HSAs in identifying blind children there is a need to compare their productivity with an alternative method in which local volunteers referred to as Key Informants (KIs) are trained.

KIs are individuals who have lived and worked in their communities and who have played vocation roles to improve the well-being of their communities. These people have an advantage in that they are familiar with the people and the local conditions that affect the community [14]. The KI method is a relatively quick and effective method of identifying blind children in the community [15] and has now been evaluated in several countries, including Malawi [14,16-22].

The current study was undertaken to compare the effectiveness of HSAs and KIs in identifying blind children after similar sessions of orientation and training.

## Methods

This was a population-based assessment of community-based case detection models that deployed and assessed the performance of trained HSAs and KIs in identifying blind children. The study was undertaken in the Mulanje district (population 435 753) in southern Malawi. The district was chosen as a pilot study because it was near the eye unit in Blantyre yet had characteristics similar to those of other rural districts in the country. The district has two hospitals, one Government, and one faith-based, and 23 health centres. At the time of the study (2007–2009) there were 240 HSAs in the district, under the management of the District Health Office, Department of Environmental Health. There was one full time paramedic ophthalmic clinical officer working at the district hospital who was mainly responsible for the community outreach eye programme.

A map of the district was obtained from the Department of Environmental Health and the district was divided into 6 clusters which consisted of well-defined geographical zones, each with a population of approximately 70 000–80 000, 42% of whom were children less than 16 years old (i.e. approximately 29 400–33 600 children per cluster). With a prevalence estimate of blindness in children of 8 per 10 000 children 0–15 years old, each cluster was expected to have approximately 24–27 blind children (144–162 in all 6 clusters). The six clusters were paired so that each pair had similar characteristics and training was randomly allocated to either HSA or KI training. In each cluster 20 HSAs or 20 KIs were selected for training.

### Selection of HSAs

A list of the names of all villages in each cluster, together with their population size and the name of the HSA responsible for each village was obtained from the District Health Office. Twenty villages were selected in each cluster using proportion probability to size procedures. A total of 60 named HSAs were invited for training.

### Selection of KIs

Each of the 60 villages was visited and the village headmen were asked to identify one volunteer (KI) using preset criteria which stated that the selected person had to be willing to be involved, had the time, could read and write and knew the village well. These individuals were invited for training.

### Training

The dates and venue for training were communicated to the selected KIs and HSAs. Training was undertaken by a team comprising a community ophthalmologist (KK), a childhood blindness coordinator, an ophthalmic clinical officer from the tertiary referral eye hospital (Lions Sight First Eye Hospital) in Blantyre, and an ophthalmic clinical officer from the district. A training curriculum/manual was developed in English and translated to the local language (Chichewa). Training for HSAs and KIs was conducted on separate consecutive days in different venues within the cluster. Training materials and methods included lectures, posters of eye conditions, flip charts, demonstration and practical of visual acuity testing in children, discussion and group work. Training emphasized the causes of blindness in children and their management, how to identify and list children they suspected of being blind, and how to identify blind children with normal appearing eyes. Blindness was defined as presenting visual acuity of <3/60 in the better eye. Low vision and visually impaired children were to be listed as not blind but as having other eye problems. HSAs and KIs were told to use multiple methods (door to door, school screening, health education, church/mosque announcements) to identify children and that they should indicate on their reporting form which method was used for each child.

The HSA training was conducted in English and took about eight hours (whole working day) while the KI training was conducted in Chichewa and lasted for five hours. The difference was because HSAs had a better educational background (secondary education) and so could understand the anatomy of the eye and which diseases could affect the eye. Most of the KIs selected had poorer education, and so less emphasis was placed on the anatomy and function of the different components of the visual system. At the end of training each individual was given a brochure that contained the key points of the training and which could be referred to when needed. After the training each group was given six weeks to identify, list and refer blind children from the allocated villages to an agreed examination centre within the community (a health centre or a school). The farthest distance that an HSA or KI had to walk to the examination centre was about 2 kilometers and they were all asked to attend the eye examination session on the scheduled days, bringing the listed children and their parent/guardian with them. Apart from transport reimbursements and a meal, no other incentives were given to the HSAs or KIs.

### Eye examination

Eye examinations were undertaken by the research team (led by an ophthalmologist) six weeks after training. HSA and KIs brought the list of children that they had identified and this was cross checked with the number of children who attended. All children who attended underwent a clinical examination and the causes of visual loss were classified using a modified version of the WHO classification of causes of blindness in children [23]. The clinical examination included measuring visual acuity with the log MAR Snellen chart, examination of anterior segments using a portable slit lamp, dilated examination of the posterior segment using a binocular indirect ophthalmoscope, and, where indicated, taking of intraocular pressure using a Perkins tonometer. Children who were listed but did not attend the examination site were traced, and examined in the community. All parents who needed their child to be referred were counseled and assured of transport reimbursements, and given a referral form to take to the eye department in Blantyre.

After the end of the examination all the HSAs and KIs who attended were invited to take part in focus group discussions to determine the challenges they had faced in identifying blind children. The HSAs/KIs who did not attend the examination session were traced and had in-depth interviews to find out why they had not attended nor brought any children. A total of six focus group discussions were conducted with the HSAs and KIs groups separately (three of each).

Ethical approval for the study was obtained from the College of Medicine Research Committee (COMREC) in Malawi and the London School of Hygiene and Tropical Medicine, United Kingdom. Informed written consent was obtained from all parents/guardians of all children who were examined and also from all HSAs and KIs who attended interviews.

## Results

A total of 6 training sessions were conducted (3 for each group), and 59 HSAs and 64 KIs were trained. The mean age of the HSAs was 29 years (range 20 to 50 years) and 49% were men. The mean age of the KIs was 35 years (range 18 to 68 years) and 53% were men. The total catchment population covered by the HSAs and the KIs were very similar, at approximately 199 500 and 197 000, respectively. According to the WHO estimates of blindness in children in this region (8 per 10 000) a total of 133 blind children would have been expected.

A total of 167 children were listed, 162 by KIs and 5 by HSAs. Of the children identified by the KIs, 155 attended the examination site as did 3 children identified by the HSAs. Three further children identified by the KIs and two by the HSAs could be traced in the community and were examined. Four children identified by the KIs could not be traced. In total 163 children were examined: 158 (97%) had been identified by the KIs. Only 22 of 163 (13.5%) children were confirmed to be blind, 20 (90.9%) of whom had been identified by the KIs. KIs, therefore, identified 10 times as many blind children as HSAs in approximately the same catchment population. The majority of the blind children were boys (N = 17, 77%) and their ages ranged from 1 to 15 years. Table 1 shows the gender distribution of children identified by HSAs and KIs, while Table 2 shows their age range. Both groups identified more boys than girls. Both blind children identified by HSAs were aged 0–5 years, while KIs identified children of all ages.

**Table 1:** Gender distribution of children identified by HSAs and KIs

	**Health Surveillance Assistants (HSAs)**				**Key Informants(KIs)**				**Total**			
	**Examined**		**Confirmed blind**		**Examined**		**Confirmed blind**		**Examined**		**Confirmed blind**	
	N	%	N	%	N	%	N	%	N	%	N	%
Boys	3	60	2	100	80	51%	15	75%	83	51%	17	77.3%
Girls	2	40	0	0	78	49%	5	25%	80	49%	5	22.7%
Total	5	100	2	100	158	100%	20	100%	163	100%	22	100.0%

**Table 2:** Age frequency distribution of children identified by HSAs and KIs

	**Health Surveillance Assistants (HSAs)**				**Key Informants(KIs)**				**Total**			
	**Examined**		**Confirmed blind**		**Examined**		**Confirmed blind**		**Examined**		**Confirmed blind**	
	N	%	N	%	N	%	N	%	N	%	N	%
0-5 years	4	80	2	100	48	30%	7	35%	52	32%	9	41%
6-10 years	1	20	0	0	50	32%	9	45%	51	31%	9	41%
11-15 years	0	0	0	0	60	38%	4	20%	60	37%	4	18%
Total	5	100	2	100	158	100%	20	100%	163	100%	22	100%

Cataract was the most common cause of blindness (50%), followed by corneal scarring (13.6%), cortical blindness (13.6%) and others (Table 3).

### Findings from group discussions

The KIs mainly used door to door visits to identify blind children whereas the HSAs used health education during immunization clinics. The KIs reported that making door to door visits was very time consuming, taking them several days. HSAs reported that they were too busy with other activities so they could not go door to door. KIs reported that they were motivated by the need to help their communities while HSAs said that they would have been more motivated if they had been given financial incentives to compensate for the extra work and time. Most KIs reported that they had visited all the villages allocated to them, but more than half of the HSAs admitted that they had not completed the job.

## Discussion

This study compared the effectiveness of using PHC workers (HSAs) with using KIs in identifying blind children in Malawi. The findings suggest that KIs are better than HSAs in identifying blind children. There are several reasons why this may be the case: HSAs reported limited time as they were engaged with other duties, whereas KIs may not have had the same time constraints; HSAs mostly used health promotion during immunization clinics while the KIs mostly went door to door. This is likely to account for an important factor since immunization clinics mainly deal with children under the age of five years and may not have much contact with older children and if there are more blind older children then HSAs would not identify them. Another factor to consider is health-seeking behavior: if parents believe there is nothing that can be done for their child then they will not seek services, and the only way these children will be identified is by visiting them within the community as done by KIs. Even then, parents may not acknowledge they have a disabled child, on account of shame or wanting to maintain privacy. The HSAs reported that they were demotivated by lack of financial incentives but this was not reported by the KIs. It should be noted that WHO guidelines on application of incentive schemes in health care acknowledges that financial incentives alone are not sufficient to retain and motivate staff [24]. Non-financial incentives play an equally crucial role in improving performance and productivity. Lack of skills was unlikely to have contributed to the difference as HSAs and KIs had similar training. Issues of limited supervision may have played a role among the HSAs, and this is supported by Muller et.al. [10], who pointed out that supportive supervision coupled with adequate training is one of the factors that could lead to increased outputs among PEC workers. Our study findings are similar to findings published by Shija et.al. [21], conducted in a rural area in Tanzania, who found that KIs identified 25 times as many blind children as PHC workers. Both these studies demonstrate the limitations in using PHC workers in PEC in Africa, a situation that may be different and acceptable in other settings.

The overall number of blind children identified (N = 22) from the entire population is much smaller than what would be anticipated using WHO prevalence estimate of blindness in children in this region [7]. The WHO prevalence estimates use under 5-year-old mortality rates as a proxy indicator, which is likely to vary between regions. However, the number identified is far below the expected number, suggesting that either both approaches (KI/HSA) do not work very well in rural Africa or that the WHO estimates are too high. Available information suggests that even though the KI method may be good for determining the causes of blindness in children, it tends to underestimate prevalence as some children are missed [25]. Unless a population based survey is done for comparison with the KI method in this region, it is difficult to determine how many children were missed by KIs. Some alternatives to determine how many children are missed would be to check records of identified children from the study area that reported at a tertiary hospital and comparing with records of children identified by KI. This approach would, however, be limited since children with non reversible causes of blindness (corneal scarring, retinal diseases) and children with multiple systemic abnormalities are unlikely to access the eye hospital.

Cataract was the most common cause of blindness in 50% of all blind children, second to cortical blindness and cornea scarring (Table 3). Available information suggests that with control of corneal blindness from vitamin A deficiency and measles, cataract in children is becoming a relatively more common cause of blindness [26]. 

**Table 3:** Anatomical causes of bilateral blindness, by sex

	**Boys**		**Girls**		**All**	**Total**
	**N**	**%**	**N**	**%**	**N**	**%**
Cataract	10	45.5	1	4.5	11	50.0
Corneal scarring	2	9.1	1	4.5	3	13.6
Cortical blindness	2	9.1	1	4.5	3	13.6
Glaucoma	2	9.1	0	0.0	2	9.1
Optic atrophy	1	4.6	1	4.6	2	9.1
Refractive error	0	0.0	1	4.6	1	4.6
Total	17	77.3	5	22.7	22	100.0

The reasons why more boys than girls were brought to the examination site in this study are likely to have several explanations. It is known that inequity in health-seeking behavior by gender contributes to boys being more likely to receive care from qualified healthcare providers than girls, resulting in girls having higher child mortality rates than boys [27]. Firstly, in the first phase of health-seeking behavior, the individual concerned or their family has to appreciate that the family member has an illness or condition that requires health care. There is some evidence that family members are less likely to perceive a girl as being unwell in developing countries than boys [28], which means that the process of decision making is not even initiated. In the case of this study, this means that parents would not have acknowledged to the KIs or the HSAs that their child had an eye or vision problem as they had not acknowledged it to themselves. Secondly, even if the family does acknowledge a problem, they may be less willing to spend their limited resources in terms of money, time, energy and opportunity costs, on health care for a girl than on a boy. Poor families are more willing to put their resources into the health of their sons because they believe that boys are likely to be their financial security for the future. Factors shown to be associated with this apparent preference are low socioeconomic status; the father’s educational status; being one of many children, and how long it takes before the development of symptoms [29]. Previous studies in Africa [30,31] have also reported this gender difference in uptake as well as in follow up of cataract services in children. Since women have a greater influence on a child’s health in most societies in Africa, engaging them in activities likely to promote eye health and prevent visual loss is likely to increase the number of girls who attend eye services [30,32].

This study poses further challenges and raises a great concern for integrating PEC into PHC care in Malawi, as due to existing Ministry of Health structures and guidelines this is being done by involving the HSAs. However, the limitations of using HSAs have been clearly shown in this study. Even though they have a major role in providing child health within the community in Malawi, adding PEC to their duties is unlikely to be successful in the long term. Available options may include training of other health centre staff such as medical assistants and nurses who regularly see adults and children of all ages; or using the midlevel ophthalmic clinical officer (OCO) from the district hospitals to provide regular satellite PEC visits within health centres. The advantage of the latter group is that they are a well trained and skilled group who are specifically trained in eye care and can offer quality services but the challenge is how to increase their numbers so that they cover entire districts on a regular basis.

Even though the study has shown that it is the KIs who have the potential to identify more blind children, issues regarding costs of maintaining the KIs and their sustainability are of major concern and need to be explored further before recommending any policy change. The findings of this study should be taken within the context of limitations of this study as among the KIs the reasons for their productivity were not explored in great depth. The focus groups only included the KIs/HSAs who attended the eye examination sessions. It is possible that those who did not attend had different reasons; therefore, the results of the findings should only be generalized to the rest of the HSAs and KIs with caution. The pre-set criteria for the identification of KIs may have resulted in selecting those with the most enthusiasm for the task while for the HSAs these were already employed.

## Conclusion

This study has shown that using HSAs for identifying blind children is not effective in comparison to using KIs. For Malawi using KIs may be an alternative for PEC; but the long term cost-benefits of using KIs versus HSAs need further exploration.

## Competing interests

The authors declare that they have no competing interests.

## Authors’ contributions

KK: conceived the study, participated in data collection and analysis and drafted the manuscript. RN: participated in data collection and entry and reviewed the article. FM: participated in data collection and reviewed the article. CG: supervised the work and edited the manuscript. All authors read and approved the final manuscript.

## References

[B1] WHO Task shifting: global recommendations and guidelines 2008 www.who.int/healthsystems/TTR-TaskShifting.pdf

[B2] Frenk J Reinventing primary health care: the need for systems integration Lancet 2009 374 170 173 10.1016/S0140-6736(09)60693-0 19439350

[B3] World Health Organization Primary Health Care. Now More Than Ever. The World Health Report WHO/PBL/90.19 2008 WHO Published by WHO. http://www.who.int/whr/2008/en/index.html

[B4] WHO The Alma-Ata conference on primary health care WHO Chron 1978 32 409 430 31034

[B5] Murthy G Raman U Perspectives on primary eye care Community Eye Health 2009 22 10 11 19506714 PMC2683556

[B6] Konyama K Essential components of primary eye care Community Eye Health 1998 11 19 21 17492026 PMC1706048

[B7] Chandna A Gilbert C When your eye patient is a child Community Eye Health 2010 23 1 3 20523854 PMC2873665

[B8] Courtright P Murenzi J Mathenge W Munana J Müller A Reaching rural Africans with eye care services: findings from primary eye care approaches in Rubavu District, Rwanda Trop Med Int Health 2010 15 692 696 10.1111/j.1365-3156.2010.02530.x 20374559

[B9] Courtright P Seneadza A Mathenge W Eliah E Lewallen S Primary eye care in sub-Saharan African: do we have the evidence needed to scale up training and service delivery? Ann Trop Med Parasitol 2010 104 361 367 10.1179/136485910X12743554760225 20819303

[B10] Muller A Murenzi J Mathenge W Munana J Courtright P Primary eye care in Rwanda: gender of service providers and other factors associated with effective service delivery Trop Med Int Health 2010 15 529 533 10.1111/j.1365-3156.2010.02498.x 20345558

[B11] WHO Strengthening Health Systems to Improve Health Outcomes: WHO’s Framework for Action 2007 Geneva http://www.who.int/healthsystems/strategy/everybodys_business.pdf

[B12] Rohde J Cousens S Chopra M Tangcharoensathien V Black R Bhutta ZA Lawn JE 30 years after Alma-Ata: has primary health care worked in countries? Lancet 2008 372 950 961 10.1016/S0140-6736(08)61405-1 18790318

[B13] Kadzandira J Chilowa W The Role of Health Surveillance Assistants (HSAs) in the Delivery of Health Services and Immunization in Malawi UNICEF Evaluation Report 2001 www.unicef.org/evaldatabase/files/MLW_01-04.pdf

[B14] Muhit MA Shah SP Gilbert CE Hartley SD Foster A The key informant method: a novel means of ascertaining blind children in Bangladesh Br J Ophthalmol 2007 91 995 999 10.1136/bjo.2006.108027 17431019 PMC1954788

[B15] Muhit M Finding children who are blind Community Eye Health 2007 20 30 31 17612695 PMC1906925

[B16] Xiao B Fan J Deng Y Ding Y Muhit M Kuper H Using key informant method to assess the prevalence and causes of childhood blindness in Xiu’shui County, Jiangxi Province, Southeast China Ophthalmic Epidemiol 2011 18 30 35 10.3109/09286586.2010.528138 21117893

[B17] Kalua K Patel D Muhit M Courtright P Productivity of key informants for identifying blind children: evidence from a pilot study in Malawi Eye (Lond) 2009 23 7 9 10.1038/eye.2008.49 18344959

[B18] Kalua K Patel D Muhit M Courtright P Causes of blindness among children identified through village key informants in Malawi Can J Ophthalmol 2008 43 425 427 10.3129/I08-084 18711455

[B19] Husain L Using the key informant method to investigate childhood blindness related to vitamin A deficiency disorder in six rural sub-districts in Bangladesh Community Eye Health 2007 20 7 8 17637851 PMC1919439

[B20] Boye J Validating key informant method in detecting blind children in Ghana J Comm Eye Health 2005 18 131

[B21] Shija F Shirima S Lewallen S Courtright P Comparing key informants to health workers in identifying children in need of surgical eye care services Int Health 2012 4 1 3 10.1016/j.inhe.2011.09.003 24030874

[B22] Razavi H Kuper H Rezvan F Amelie K Mahboobi-Pur H Oladi MR Muhit M Hashemi H Prevalence and causes of severe visual impairment and blindness among children in the Lorestan province of Iran, using the key informant method Ophthalmic Epidemiol 2010 17 95 102 10.3109/09286581003624954 20302431

[B23] Gilbert C Foster A Négrel AD Thylefors B Childhood blindness: a new form for recording causes of visual loss in children Bull World Health Organ 1993 71 485 489 8261552 PMC2393473

[B24] WHO Guidelines Incentives for Heath Professionals. Global Health Workforce alliance, in WHO: Global Health Workforce alliance International Council of Nurses, International Hospital; Federation, International Pharmaceutical Federation, World Confederation for; Physical Therapy, World Dental Federation, World Medical Association 2008 1 44 http://www.who.int/workforcealliance/documents/Incentives_Guidelines%20EN.pdf

[B25] Gona JK Xiong T Muhit MA Newton CR Hartley S Identification of people with disabilities using participatory rural appraisal and key informants: a pragmatic approach with action potential promoting validity and low cost Disabil Rehabil 2010 32 79 85 10.3109/09638280903023397 19925280 PMC3166842

[B26] Gilbert C Muhit M Twenty years of childhood blindness: what have we learnt? Community Eye Health 2008 21 46 47 19030129 PMC2580065

[B27] Najnin N Bennett CM Luby SP Inequalities in care-seeking for febrile illness of under-five children in urban Haka, Bangladesh J Health Popul Nutr 2011 29 523 531 22106759 10.3329/jhpn.v29i5.8907 PMC3225115

[B28] Pokhrel S Sauerborn R Household decision-making on child health care in developing countries: the case of Nepal Health Policy Plan 2004 19 218 233 10.1093/heapol/czh027 15208278

[B29] Tursz A Crost M An epidemiologic study of health care seeking behavior of children under 5 years of age by sex in developing countries Rev Epidemiol Sante Publique 1999 47 S133 S156 10575716

[B30] Bronsard A Shirima S Cataract surgery: ensuring equal access for boys and girls Community Eye Health J 2009 22 28 29 PMC2760280 19888368

[B31] Mwende J Bronsard A Mosha M Bowman R Geneau R Courtright P Delay in presentation to hospital for surgery for congenital and developmental cataract in Tanzania Br J Ophthalmol 2005 89 1478 1482 10.1136/bjo.2005.074146 16234457 PMC1772945

[B32] Kothari G Working with women to improve child and community eye health Community Eye Health 2009 22 20 21 19888363 PMC2760275

